# PGC1*α* Activators Mitigate Diabetic Tubulopathy by Improving Mitochondrial Dynamics and Quality Control

**DOI:** 10.1155/2017/6483572

**Published:** 2017-03-20

**Authors:** So-Young Lee, Jun Mo Kang, Dong-Jin Kim, Seon Hwa Park, Hye Yun Jeong, Yu Ho Lee, Yang Gyun Kim, Dong Ho Yang, Sang Ho Lee

**Affiliations:** ^1^Division of Nephrology, Department of Internal Medicine, CHA Bundang Medical Center, CHA University, Seongnam, Republic of Korea; ^2^Division of Nephrology, Department of Internal Medicine, Kyung Hee University Hospital at Gangdong, Kyung Hee University, Seoul, Republic of Korea

## Abstract

*Purpose.* In this study, we investigated the effect of PGC1*α* activators on mitochondrial fusion, fission, and autophagic quality control in renal tubular cells in a diabetic environment in vivo and in vitro. We also examined whether the upregulation of PGC1*α* attenuates diabetic tubulopathy by normalizing mitochondrial homeostasis.* Methods*. HKC8 cells were subjected to high-glucose conditions (30 mM D-glucose). Diabetes was induced with streptozotocin (STZ, 50 mg/kg i.p. for 5 days) in male C57/BL6J mice. AICAR or metformin was used as a PGC1*α* activator.* Results.* Treatment with the PGC1*α* activators AICAR and metformin improved functional mitochondrial mass in HKC8 cells in high-glucose conditions. Moreover, in renal proximal tubular cells, increased PGC1*α* activity correlated with the reversal of changes in Drp1, Mfn1, and LC3-II protein expression in a high-glucose environment. Normalized mitochondrial life cycles resulted in low ROS production and reduced apoptosis. AICAR and metformin treatment effectively mitigated albuminuria and renal histopathology and decreased the expression of TGF*β*1 and *α*SMA in the kidneys of diabetic mice.* Conclusions*. Our results demonstrate that increases in PGC1*α* activity improve diabetic tubulopathy by modulating mitochondrial dynamics and autophagy.

## 1. Introduction

The global prevalence of diabetes among adults over 18 years of age has rapidly risen from 4.7% in 1980 to 8.5% in 2014 [1]. Diabetic kidney disease (DKD) is the leading cause of end-stage renal disease, which requires renal replacement therapy, and approximately 30% of diabetic patients have decreased renal function as a long-term complication of diabetes [2]. Moreover, DKD accounts for a significant increase in morbidity and mortality in patients with diabetes, and care for these patients is associated with substantial costs to society [3].

Although the roles of the glomerular filtration barrier in the development of albuminuria, which is a cardinal feature of DKD, are known, recent data have shown that only selective defects in tubular transport processes result in albuminuria or proteinuria [4]. Furthermore, it is clear that tubular epithelial cells under high-glucose conditions show elevated activation of proinflammatory and profibrotic signal transduction pathways, which contribute to progressive interstitial fibrosis [5]. Given that the extent of renal dysfunction correlates well with the degree of interstitial fibrosis [6], diabetic tubulopathy must be considered and explored as much as diabetic glomerulopathy in the pathogenesis of DKD.

Mitochondrial dysfunctions or abnormalities are associated with DKD, but the mechanistic nature of this association needs to be elucidated [7–12]. PGC1*α* (peroxisome proliferator-activated receptor gamma coactivator 1-alpha) is a transcriptional coactivator that regulates the genes involved in energy metabolism, including mitochondrial-associated proteins and transcriptional factors [13]. Interestingly, reduced PGC1*α* expression in diabetic kidneys indicates that PGC1*α* may mediate mitochondrial abnormalities during the development and progression of DKD [13].

In this study, we demonstrate that reduced PGC1*α* expression in renal proximal tubular cells is related to abnormal dynamics and quality control of mitochondria under high-glucose conditions and that PGC1*α* activation attenuates the effect of high-glucose conditions on kidney tubules in vivo and in vitro.

## 2. Materials and Methods

### 2.1. Cell Culture

The human renal proximal tubular epithelial cell line HKC8 was obtained from Dr. L. Rausen (Johns Hopkins University, Baltimore, MD) and was maintained in Dulbecco's Modified Eagle Medium supplemented with Ham's F12 medium (DMEM/F12; Invitrogen). DMEM/F12 was supplemented with 10% fetal bovine serum and 1% penicillin/streptomycin (WelGENE, Daegu, Republic of Korea). Before experiments, cells were maintained in medium containing 5–30 mM D-glucose. To increase PGC1*α* activity, 1 mM 5-aminoimidazole-4-carboxamide ribonucleotide (AICAR; Sigma-Aldrich St. Louis, MO, USA) or 1 mM metformin (Sigma-Aldrich, St. Louis, MO, USA) was added to the culture media.

### 2.2. Animal Studies

Diabetes was induced in eight-week-old male C57/BL6J mice (Center for Research Animals, Seoul, Korea) by intraperitoneal injection of streptozotocin (STZ; Sigma Chemicals, Missouri) at a dose of 50 mg/kg for 5 consecutive days. In the intervention study, four groups of mice (*n* = 5) were used for three separate experiments: (1) normal control, (2) diabetic control, (3) diabetes + AICAR (300 mg/kg), and (4) diabetes + metformin (150 mg/kg). AICAR (Toronto Research Chemical Inc., Toronto, Canada) and metformin (Sigma-Aldrich, St. Louis, MO, USA) were dissolved in PBS and administered to mice via daily intraperitoneal injection with the indicated dose from 2 weeks after STZ administration for a period of 14 weeks. All mice were sacrificed 16 weeks after STZ administration, and their kidney tissues were collected for analyses.

During the experiments, body weights and serum glucose concentrations were measured weekly. All experiments were performed according to the guidelines of the Gangdong Animal Research Ethics Committee of Kyung Hee University Hospital.

### 2.3. Isolation of Total RNA, Real-Time Polymerase Chain Reaction (PCR)

Total RNA was extracted from kidney tissue using the Total RNA Isolation Kit (Macherey-Nagel, Duren, Germany) according to the manufacturer's instructions. Real-time PCR was performed using SYBR Green PCR Master mix (Fast Start Universal SYBR Green Master; Roche). To quantify the mtDNA/gDNA ratio, qPCR was used to amplify one gene from the mitochondrial genome (Nd1 in humans) and one gene from the nuclear genome (*β*-globin in humans). Primer sequences were as follows: Nd1 forward 5′-CAA ACC GGG CCC CCT TCG AC-3′; Nd1 reverse 5′-CGA ATG GGC CGG CTG CGT AT-3′; *β*-globin forward, 5′-GAG AAT GGG AAG CCG AAC ATA-3′; *β*-globin reverse 5′-CCG TTC TTC AGC ATT TGG ATT T-3′.

### 2.4. Western Blot Analysis

Cells and kidney tissues were washed with PBS and lysed in ice-cold lysis buffer containing a protease inhibitor cocktail (Roche Diagnostics, Mannheim, Germany). Proteins were separated with 8–15% SDS-PAGE and then transferred onto a polyvinylidene difluoride membrane (Millipore, Madrid, Spain) by electroblotting. The membrane was blocked for 1 h at room temperature and then incubated overnight at 4°C with anti-PGC-1*α*, anti-Bax, anti-cytochrome C, anti-E-cadherin (1 : 1000, Santa Cruz, USA), anti-phospho-PGC-1*α* (R&D system Inc. Minneapolis, MN), anti-AMPK, anti-phospho-AMPK, anti-TGF-*β*, anti-Bcl2 (1 : 1000, Cell Signaling Technology, MA, USA), and *α*-SMA (1 : 1000 Abcam Inc. Cambridge, UK) primary antibodies. Subsequently, the membranes were stained with horseradish peroxidase-conjugated goat anti-rabbit or mouse immunoglobulin G (1 : 2,000, Santa Cruz CA, USA). The immunoreactive bands were detected by chemiluminescence (enhanced chemiluminescence; BioFX Laboratories, Inc., Maryland). GAPDH (1 : 2,000, Santa Cruz, CA, USA) was used as an internal control.

### 2.5. Measurement of Reactive Oxygen Species (ROS)

To assess intracellular and mitochondrial ROS production, cells were incubated with H2-DCFDA and MitoSOX (Life Technologies, Seoul, Republic of Korea), after which they were examined by confocal microscopy (LSM-700; Carl Zeiss, Jena, Germany). Flow cytometry was performed to quantify the amount and change of ROS production according to manufacturer's instruction. Briefly, prepared HKC8 cells were incubated with MitoSOX Red (3 *μ*M, Life Technologies, Seoul, Republic of Korea) at 37°C for 10 minutes. Using a flow cytometer, MitoSOX Red was excited at 488 nm and fluorescence emission at 575 was measured. The cells were analysed with a FACSCalibur flow cytometry system (BD Biosciences, CA, USA), and data analysis was performed using FlowJo software version 10 (TreeStar, OR, USA). Relative fluorescence intensity was used as measurement of mitochondrial superoxide production.

### 2.6. Immunofluorescence

Cells were fixed with paraformaldehyde, permeabilized, blocked with bovine serum albumin (BSA), and incubated with primary antibodies. After washing with PBS, the samples were reincubated with secondary antibodies conjugated with Alexa Fluor 488 or 594 (Life Technologies, Korea).

For immunofluorescence of kidney tissues, 4 *μ*m thick cryostat sections were prepared, mounted on glass slides, air-dried, and rehydrated with PBS. Similarly, 3 *μ*m thick paraffin-embedded sections were prepared, dewaxed, and rehydrated. After blocking with BSA, sections were incubated with primary antibodies and then Alexa Fluor-conjugated secondary antibodies.

Cells and tissues were counterstained with DAPI to delineate the nuclei, and the sections were examined by confocal microscopy (LSM-700; Carl Zeiss, Jena, Germany).

### 2.7. Assessment of Renal Tissue Morphology

Four-micrometer-thick paraffin-embedded sections of kidneys from the four mouse groups were dewaxed in xylene and rehydrated. For the histological assessment of tubulointerstitial injury and fibrosis, the sections were stained with Periodic acid-Schiff or Masson's trichrome reagents. Twenty high-power fields of corticomedullary areas in each section were randomly selected for analysis of tubulointerstitial injury and fibrosis, respectively, and were blindly scored by an independent pathologist. The total tubular injury was graded on a scale of 0 to 3 based on the percentage of normal tubules and the amount of tubular dilatation and tubular epithelial cell destruction as follows: 0, absent; 1, 1–25%; 2, 26–50%; 3, >50% of the tubular area affected [14, 15]. Fibrosis was expressed as a percentage of positive area (twenty) among total field using computer-assisted image analysis [16].

### 2.8. Electron Microscopy Analysis

Renal cortices were minced into 1 mm^3^ pieces and processed for electron microscopy. Thin sections were prepared for electron microscopy to delineate the extent of mitochondrial fragmentation in the tubules [17]. Mitochondria longer than 2 *μ*m were considered filamentous, and those shorter than 0.5 *μ*m with a spherical configuration were considered fragmented.

### 2.9. Statistics

The data are expressed as the means ± SEMs. The results were analysed using a Kruskal-Wallis nonparametric test for multiple comparisons. Significant differences in the Kruskal-Wallis test were confirmed by the Wilcoxon rank-sum and Mann–Whitney* U* tests (used to compare mean differences). *p* values < 0.05 were considered to be statistically significant. All of the analyses were completed using SPSS software (version 22; SPSS, Chicago, IL, USA) for Windows.

## 3. Results

### 3.1. Decreased Expression of PGC1*α* and AMPK in Renal Proximal Tubular Cells Subjected to High-Glucose Conditions

**Figure 1:**
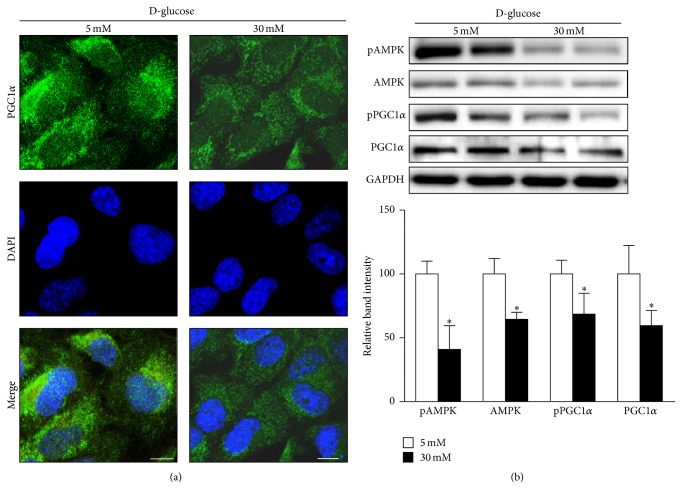
PGC1*α* and AMPK expression in human renal proximal tubular cells (RPTCs) under high-glucose conditions. (a) Representative confocal fluorescence images of PGC1*α* indicate that high-glucose conditions suppress PGC1*α* expression in human RPTCs. (b) Western blot analyses reveal downregulation of PGC1*α* and AMPK under high-glucose conditions (^*∗*^*p* < 0.05 compared with the 5 mM glucose condition, scale bar 10 *μ*m).

We studied the effect of a high-glucose environment on the expression of the key transcriptional coactivator of mitochondrial proteins, PGC1*α*, in human renal proximal tubular cells after treatment with 5 mM (low) or 30 mM (high) D-glucose for 24 h. Immunohistochemistry revealed that PGC1*α* expression after high-glucose treatment was lower than that observed after low-glucose treatment in HKC8 cells (Figure 1(a)). Consistently, Western blot analysis showed decreased PGC1*α* expression and phosphorylation, as well as decreased expression of phosphorylated AMP-activated protein kinase (AMPK) and total AMPK, in samples subjected to high-glucose conditions (Figure 1(b)). AMPK is an important regulator of cellular metabolism, and direct phosphorylation of PGC1*α* by AMPK increases PGC1*α* transcriptional activity [18]. These findings suggest that suppression of AMPK activity is linked with low PGC1*α* expression levels in response to high concentrations of glucose in human renal proximal tubule cells.

### 3.2. Alterations in Mitochondrial Dynamics, Autophagy, ROS Production, and Apoptosis in Renal Proximal Tubular Cells Subjected to High-Glucose Conditions

We hypothesized that the suppression of PGC1*α* in human renal proximal tubular cells by a high-glucose environment (30 mM) would change mitochondrial homeostasis.

**Figure 2:**
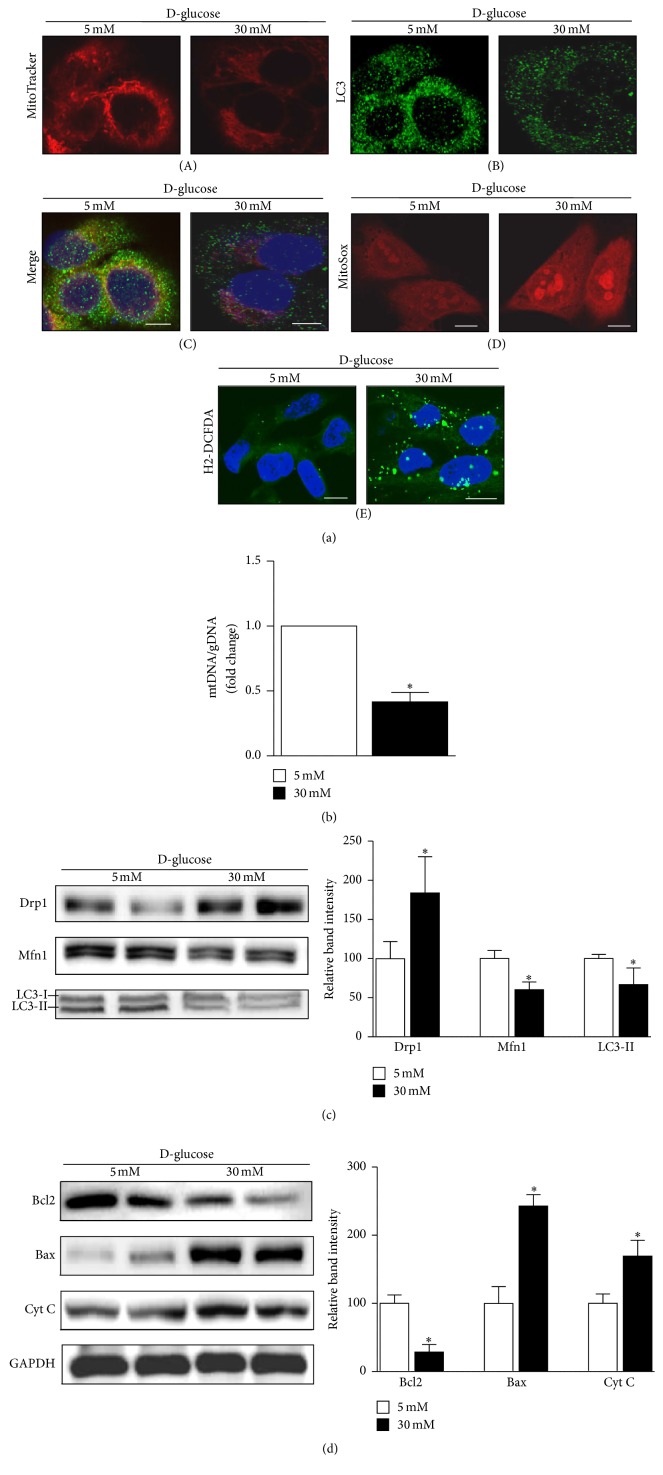
Effect of high-glucose (HG) conditions on mitochondrial dynamics, autophagy, apoptosis, and ROS production in human renal proximal tubular cells (RPTCs). (a, A, B, and C) Under high-glucose conditions, the number of MitoTracker Red-labeled mitochondria and the expression of LC3, an autophagy marker, decrease. (a, D and E) However, mitochondrial and cytosolic ROS increase, as detected by MitoSOX (red) or H2-DCFDA (green) staining. (b) Decreased mitochondrial mass, as assessed by the mtDNA/gDNA ratio, in human RPTCs under high-glucose condition. (c) Western blot analyses reveal upregulation of the mitochondrial profission protein Drp1; the profusion protein Mfn1 and the autophagy-related protein LC3II were downregulated. (d) Bax and cytochrome C, apoptosis regulatory proteins, were upregulated in a HG environment, whereas Bcl2, an antiapoptotic protein, was downregulated (^*∗*^*p* < 0.05 versus the 5 mM glucose condition; scale bar 10 *μ*m).

MitoTracker Red labels functioning mitochondria with intact membrane potentials within cells. Confocal microscopy indicated a decreased amount of MitoTracker Red-positive, functioning mitochondria in cells exposed to a high-glucose environment (Figure 2(a)(A)). Mitochondrial content was also estimated based on the ratio of mitochondrial to genomic DNA (mtDNA/gDNA). These analyses showed significantly reduced mitochondrial content in HKC8 cells exposed to high-glucose concentrations (Figure 2(b)).

Immunoblot analyses revealed upregulation of mitochondrial profission protein Drp1, whereas profusion protein Mfn1 was downregulated in HKC8 cells in a high-glucose environment (Figure 2(c)). Drp1 is a member of the dynamin family of large GTPases and controls the final step in mitochondrial fission [19]. Therefore, increased expression of Drp1 protein indicates excessive mitochondrial fragmentation, which could occur before cell injury [20]. LC3 was used as an autophagy marker, and after exposure to the high-glucose environment, the intensity of LC3 fluorescence was reduced (Figure 2(a)(B)). When autophagy is initiated, a cytosolic form of LC3 (LC3-I) is converted to LC3-II through conjugating to phosphatidylethanolamine; then elongated double membranes can encase cytoplasmic component forming autophagosome. Therefore, the amount of LC3-II implicates autophagosomal activities. As detected by immunoblot analyses, the expression level of LC3-II was lower in a high-glucose environment (Figure 2(c)). Moreover, confocal microscopy analysis of a combination of LC3 and MitoTracker Red staining revealed that the removal of damaged or redundant mitochondria by autophagy was reduced in the high-glucose environment (Figure 2(a)(C)).

In addition, the apoptogenic proteins cytochrome C (Cyt C) and Bax were upregulated and the antiapoptotic protein Bcl2 was downregulated in human renal proximal tubular cells exposed to high concentrations of glucose (Figure 2(d)). Increased mitochondrial and cytosolic ROS generation was observed in cells subjected to high-glucose conditions as determined by a higher intensity staining with MitoSOX and H2-DCFDA probes (Figure 2(a)(D and E)).

These data suggest that renal proximal tubular cells exposed to a high-glucose environment experience a decrease in the amount of functional mitochondria and impaired mitochondrial dynamics and quality control. Concomitantly, increased ROS leakage from dysfunctional and fragmented mitochondria contribute to increased apoptosis in cells exposed to high-glucose environments.

### 3.3. AICAR or Metformin Attenuates the Effect of High-Glucose Concentrations on Mitochondrial Dynamics and Autophagy in Renal Proximal Tubular Cells by PGC1*α* Activation

**Figure 3:**
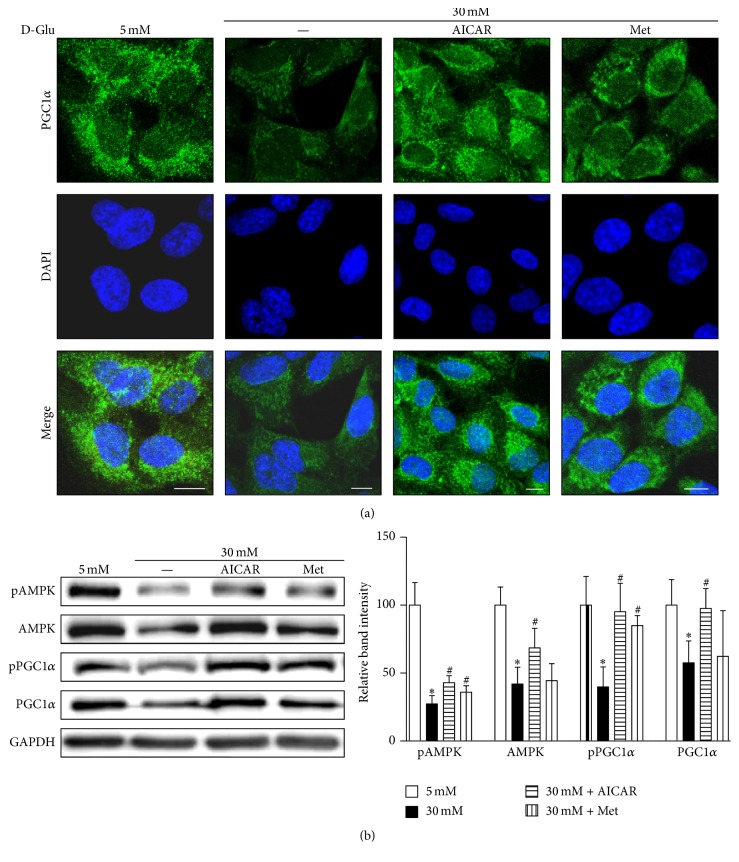
AICAR and metformin protect against the decrease in AMPK and PGC1*α* expression under high-glucose (HG, 30 mM) concentrations in human renal proximal tubular cells. (a) Representative confocal fluorescence images of PGC1*α* indicate that AICAR or metformin restore PGC*α* activity in human RPTCs subjected to HG concentrations. (b) Immunoblotting results show that increased AMPK activity and restored PGC1*α* expression are observed in human RPTCs subjected to high glucose and AICAR or metformin treatment (^*∗*^*p* < 0.05 versus 5 mM glucose treatment; ^#^*p* < 0.05 versus 30 mM glucose treatment; scale bar 10 *μ*m).

To examine whether changes in PGC1*α* expression restore the mitochondrial functionality in renal proximal tubular cells exposed to high-glucose concentrations, we treated HKC8 cells with AICAR or metformin (PGC1*α* activators through AMPK phosphorylation) for 24 h. Figures 3(a) and 3(b) show that increased AMPK activity restores PGC1*α* expression in the human renal proximal tubular cells exposed to high-glucose concentrations after either AICAR or metformin treatment.

**Figure 4:**
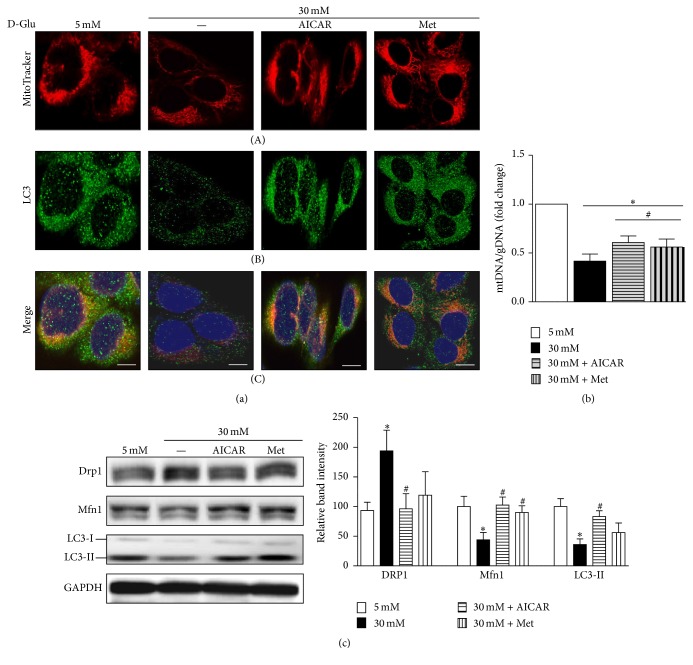
AICAR and metformin ameliorate the effect of high-glucose (HG) concentrations on mitochondrial dynamics and autophagy in human renal proximal tubular cells (RPTCs). (a, A, B, and C) Representative confocal fluorescence images of MitoTracker and LC3 show an increase in functional mitochondria and autophagic activity after exposure to HG and treatment with AICAR or metformin in human RPTCs. (b) Increased mtDNA to gDNA ratio was observed in human RPTCs treated with metformin or AICAR in the presence of HG. (c) Western blot analyses showed the reversal of alterations in the expression levels of profission (Drp1), profusion (Mfn1), and autophagy-related proteins (LC3-II) in the presence of HG after treatment with AICAR or metformin. (^*∗*^*p* < 0.05 versus 5 mM glucose; ^#^*p* < 0.05 versus 30 mM glucose; scale bar 5 *μ*m).

Confocal microscopy also showed an increase in the number of functioning mitochondria (Figure 4(a)(A)). The ratio of mtDNA to gDNA in cells treated with metformin or AICAR in a high-glucose environment was increased, suggesting the restoration of mitochondrial mass (Figure 4(b)). Autophagic activity for the removal of dysfunctional mitochondria, detected by a combination of LC3 and MitoTracker staining, also showed recovery upon treatment with either AICAR or metformin in the high-glucose environment (Figure 4(a)(C)).

Restoration of PGC1*α* expression by AICAR or metformin treatment is accompanied by decreased expression of Drp1, whereas the levels of Mfn1 were increased even in high-glucose conditions (Figure 4(c)), suggesting reduced mitochondrial fission. During autophagy, LC3-I is converted to LC3-II, and the amount of LC3-II correlates with the number of autophagosomes. Figure 4(c) showed increased LC3-II protein expression following the treatment with high glucose implicating recovery of autophagic activities.

Excessive mitochondrial fission and decreased removal of damaged mitochondria can have negative effects on mitochondrial function and are related to Parkinson's disease and acute kidney injury [20, 21]. Our results suggest that stimulating mitochondrial biogenesis via PGC1*α* may compensate for the deleterious effects on mitochondrial dynamics and quality control under high-glucose conditions in renal proximal tubular cells.

### 3.4. AICAR and Metformin Reduce ROS Production, Apoptosis, and Expression of TGF*β*1 and *α*SMA in Renal Proximal Tubular Cells in a High-Glucose Environment

**Figure 5:**
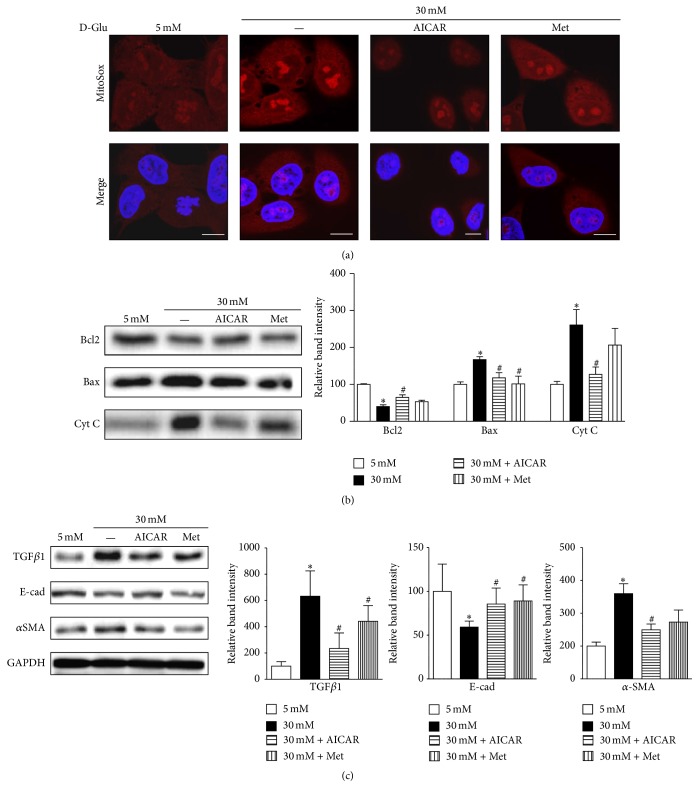
AICAR and metformin reduce ROS production and apoptosis and decrease TGF*β*1 and *α*SMA expression in human renal proximal tubular cells (RPTCs) exposed to high glucose (HG). (a) Representative confocal images of MitoSOX indicate a reduction in mitochondrial ROS production after treatment with AICAR or metformin in human RPTCs subjected to HG. (b) Western blot analyses reveal decreased expression of the apoptogenic proteins cytochrome C and Bax and increased expression of the antiapoptotic protein Bcl2 after AICAR or metformin treatment under HG conditions. (c) Western blot analyses show that AICAR or metformin treatment induces the downregulation of TGF*β*1 and *α*SMA, but upregulation of E-cad under HG conditions in human RPTCs (^*∗*^*p* < 0.05 versus 5 mM glucose; ^#^*p* < 0.05 versus 30 mM; scale bar 10 *μ*m).

Next, we examined mitochondrial ROS production and apoptosis-related protein expression to explore the effect of recovered mitochondrial homeostasis in renal proximal tubular cells treated with high-glucose concentrations. We identified decreased mitochondrial ROS production as suggested by the reduced intensity of MitoSOX staining in HKC8 cells after AICAR and metformin treatment in the presence of high-glucose concentrations (Figure 5(a) and supplementary Figure 1 in Supplementary Material available online at https://doi.org/10.1155/2017/6483572). Concurrently, immunoblot analysis showed that expression of Bcl2 was higher and the expressions of Bax and Cyt C protein were lower in renal proximal tubular cells incubated with PGC1*α* activators than those in cells subjected to high-glucose conditions (Figure 5(b)).

Notably, AICAR and metformin each induced the downregulation of TGF*β*1, whereas TGF*β*1 expression was upregulated in response to high-glucose concentrations (Figure 5(c)). TGF*β*1 is a central cytokine in renal fibrosis and has multiple functions in renal inflammation and apoptosis [22]. PGC1*α* activator treatment decreased the expression of *α*SMA induced by high-glucose concentrations. Instead, E-cadherin expression, which is a marker of epithelial phenotype, was restored by PGC1*α* activators (Figure 5(c)).

These data show that increased PGC1*α* activation in cells exposed to high-glucose concentrations restores mitochondrial abnormalities, including disturbed mitochondrial dynamics and autophagic removal. In addition, treatment of renal proximal tubular cells exposed to a high-glucose environment with PGC1*α* activators reduced mitochondrial ROS generation, apoptosis, and TGF*β*1 and *α*SMA expression.

### 3.5. Ameliorative Effect of AICAR or Metformin on Albuminuria and Renal Morphologic Characteristics by PGC1*α* Activation in STZ-Induced Diabetes in Mice

**Figure 6:**
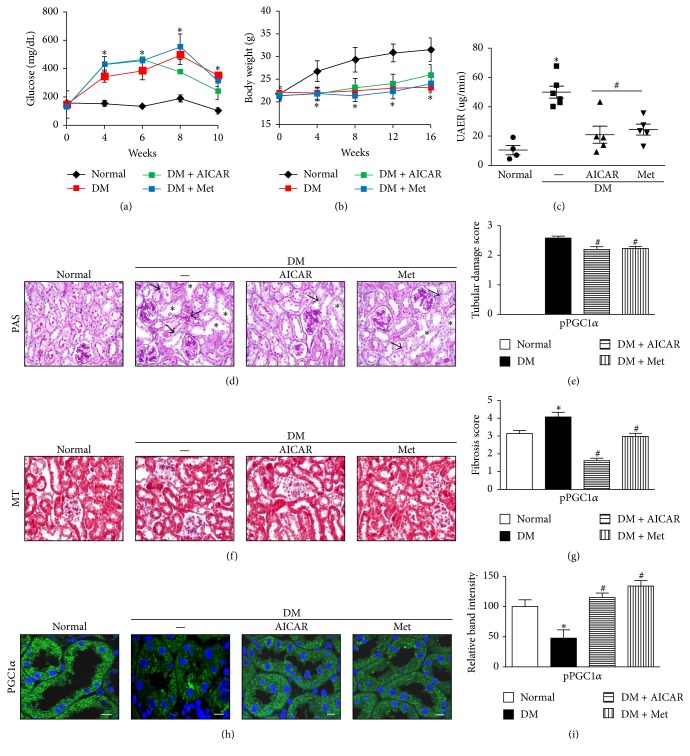
AICAR and metformin increase PGC1*α* expression and ameliorate albuminuria and renal morphologic characteristics in kidneys of streptozotocin- (STZ-) induced diabetic mice. (a and b) The STZ-induced diabetes group had higher blood glucose levels and reduced body weights compared with the normal control group. (c) The STZ-induced diabetic mice treated with AICAR or metformin exhibited a significant reduction in urine albumin excretion rate (UAER). (d and e) Tubular dilatation (asterisk) and tubular epithelial disruption (arrow) were observed in the diabetic control group. Treatment of STZ-induced diabetic mice with AICAR or metformin resulted in less cellular disruption. (f and g) Representative photographs of Masson's trichrome-stained kidneys showed decreased renal fibrotic lesions in both AICAR and metformin-treated groups compared with the diabetic control group. (h and i) Restored PGC1*α* activity was confirmed by immunofluorescence and immunoblot analysis in diabetic kidneys treated with AICAR or metformin (^*∗*^*p* < 0.05 versus normal; ^#^*p* < 0.05 versus diabetic control; scale bar 10 *μ*m).

To determine the effect of AICAR or metformin on DKD in vivo, we administered AICAR or metformin to STZ-induced diabetic mice for a period of 14 weeks. AICAR or metformin treatment in STZ-induced diabetic mice did not lower blood glucose levels (Figure 6(a)). In addition, STZ-induced diabetic mice had lower body weights compared with normal mice regardless of AICAR or metformin treatments (Figure 6(b)). However, the STZ-induced diabetic mice exhibited a significant reduction in the rate of urine albumin excretion (Figure 6(c)).

Morphology studies using PAS staining revealed tubular dilatation and tubular epithelial disruption in the diabetic control group. However, the diabetic mice treated with AICAR or metformin showed improvements in the tubular compartment with less cellular disruption (Figure 6(d), supplementary Figure 2). Semiquantitative analysis based on damage scores indicated that both AICAR and metformin treatment groups had significant decreases in tubular damage compared with the STZ group (Figure 6(e)). Analysis of Masson's trichrome staining showed a decrease in the number of renal fibrotic lesions in the AICAR and metformin-treated group compared with the diabetic group (Figure 6(f), supplementary Figure 2). Semiquantitative analysis of Masson's trichrome-stained kidneys revealed significantly lower fibrosis scores in the kidneys from diabetic mice treated with AICAR or metformin (Figure 6(g)).

Restored PGC1*α* activity in the kidneys from diabetic mice treated with AICAR or metformin was confirmed by immunofluorescence and immunoblot analyses (Figures 6(h) and 6(i) and supplementary Figure 3).

As illustrated by these results, AICAR and metformin ameliorate tubular injury and interstitial fibrosis in STZ-induced diabetic kidneys in vivo.

### 3.6. AICAR and Metformin Ameliorate Abnormal Mitochondrial Dynamics and Increase the Autophagic Removal of Mitochondria in the Tubules of STZ-Induced Diabetic Mice

**Figure 7:**
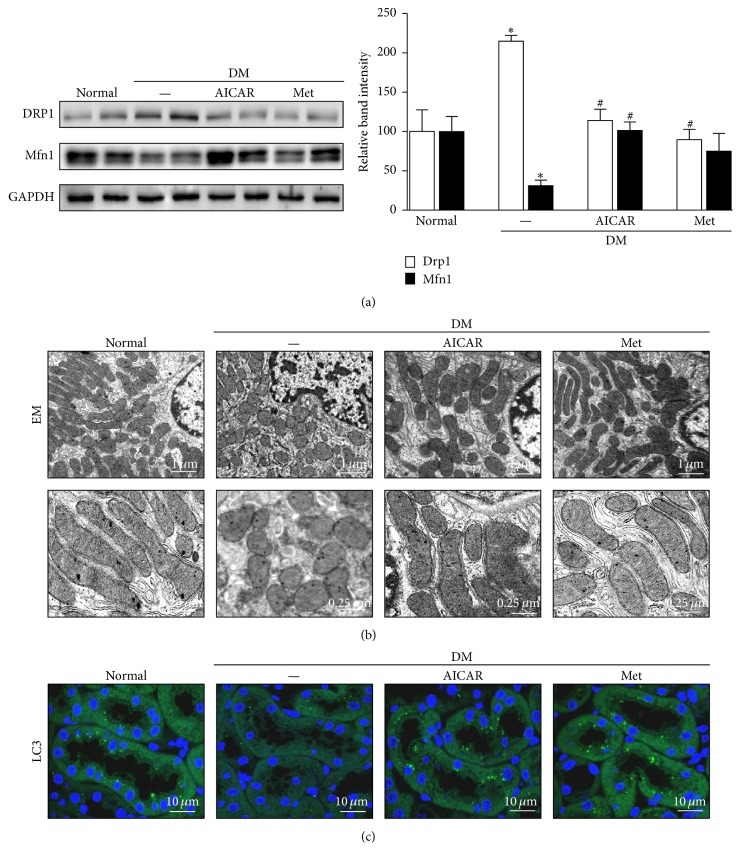
Restoration of altered mitochondrial dynamics and autophagy in STZ-induced diabetic mice after treatment of AICAR or metformin. (a) Western blot analysis revealed that AICAR or metformin treatment reverses the changes in the expression of Drp1 and Mfn1 in diabetic kidneys. (b) Electron micrographs of mitochondria in renal tubular cells. The control group displayed elongated mitochondria, whereas the diabetic group displayed short or spherical shaped mitochondria. The administration of AICAR or metformin markedly attenuated mitochondrial fragmentation in the renal tubular cells of diabetic kidneys. (c) The basal level of autophagy, indicated by punctate LC3 staining, was seen in the renal tubules of the control group and was decreased in the tubules of the diabetic group. Notably, autophagic activity was restored in the AICAR and metformin groups (^*∗*^*p* < 0.05 versus normal; ^#^*p* < 0.05 versus diabetic control).

We assessed the effect of AICAR and metformin on mitochondrial homeostasis and quality control in diabetic kidneys. The expression of Drp1, a profission protein, was increased, and the expression of Mfn1, a profusion protein, was decreased in kidneys from diabetic mice that were not treated with PGC1*α* activator (Figure 7(a)). AICAR or metformin treatment restored the expression of these mitochondrial fusion/fission proteins to levels similar to those in kidneys from the control group of mice (Figure 7(a)) accompanied by alterations in the mitochondrial morphology in the renal tubules as observed by electron microscopy (Figure 7(b)). Elongated mitochondria were observed in renal tubular cells from the control group, whereas most mitochondria from the STZ group showed short or spherically shaped mitochondria. AICAR or metformin treatment markedly attenuated mitochondrial fragmentation in renal tubular cells in the diabetic group (Figure 7(b)).

In addition, the restoration of autophagic activity was noted in the kidneys of diabetic mice treated with either AICAR or metformin as evidenced by an increase in the intensity of LC3 immunofluorescence (Figure 7(c)).

AICAR and metformin exerted an inhibitory effect against the disruption of mitochondrial dynamics and homeostasis in the renal tubules of mice with STZ-induced diabetes.

### 3.7. AICAR and Metformin Improve Renal Expression of Apoptogenic and Fibrotic Proteins in STZ-Induced Diabetic Mice

Improvement of mitochondrial quality control as a result of treatment with PGC1*α* activators may provide favorable conditions to renal tubular cells under diabetic conditions with low apoptogenic protein expression [23]. Consequently, AICAR or metformin treatment could prevent the progression of chronic kidney disease in diabetes. Stimulated renal tubule cells release the profibrotic cytokine TGF*β*1, and the activation of *α*SMA-positive myofibroblasts results in matrix production, culminating in interstitial fibrosis, which was seen in all progressive kidney diseases, including DKD [22].

**Figure 8:**
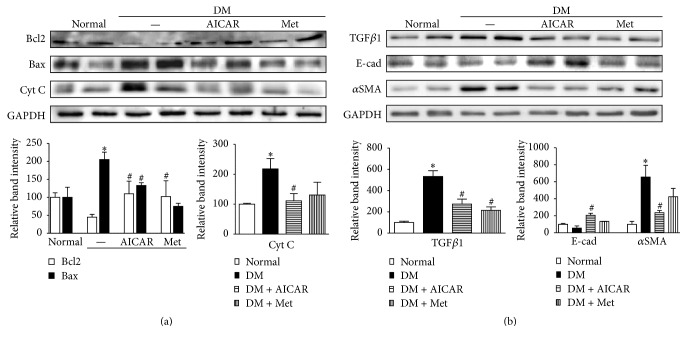
PGC1*α* activators attenuate renal expression of apoptogenic and fibrotic proteins in STZ-induced diabetic mice. (a) In diabetic kidneys, the protein expression of the antiapoptotic protein Bcl2 was markedly decreased, whereas the expression of the apoptogenic proteins Bax and cytochrome C (Cyt C) increased greatly. PGC1*α* treatment reversed the expression of these apoptogenic-related proteins. (b) Increased expression levels of TGF*β*1 and *α*SMA were seen in the kidneys from the diabetic group, and reduced expression levels of these proteins were seen in the AICAR- and metformin-treated groups. (^*∗*^*p* < 0.05 versus normal; ^#^*p* < 0.05 versus diabetic control).

To explore whether AICAR or metformin treatment could affect the expression of apoptosis-related proteins in the kidneys of STZ-induced diabetic mice, we examined the expression levels of Bcl2, Bax, and Cyt C in the kidneys from the normal control, diabetic control, AICAR-treated, and metformin-treated groups. In kidneys from the diabetic groups, the protein expression of Bcl2 was markedly decreased, and the expression of Bax was greatly increased, suggesting that a diabetic environment leads to the apoptosis of kidney cells. However, AICAR and metformin treatment reversed the expression changes of Bcl2 and Bax (Figure 8(a)). The increased expression of Cyt C in diabetic kidneys implies that Cyt C, which is a component of the mitochondrial inner membrane, is associated with tubular cell apoptosis in a diabetic environment (Figure 8(a)).

We further investigated whether AICAR or metformin could regulate TGF*β*1 and *α*SMA expression in diabetic kidneys. Both AICAR and metformin effectively lowered the protein expression levels of profibrotic cytokine TGF*β*1 and the myofibroblast marker *α*SMA in diabetic kidneys relative to their expression levels in untreated diabetic kidneys (Figure 8(b)).

## 4. Discussion

Since mitochondria were first reported as tiny intracellular organelles in the 1800s, many studies have focused on elucidating their functions in ATP production, storage of calcium ions, and regulation of apoptosis [24–26]. Moreover, the dynamic nature of mitochondria, including fission, fusion, and autophagy, has recently been elucidated [27, 28], and the abnormal process of mitochondrial turnover has been reported in various diseases [29]. Here, we show that the upregulation of PGC1*α* corrected abnormal mitochondrial dynamics and quality control in renal proximal tubular cells subjected to a high-glucose environment and effectively reduced albuminuria and tubulointerstitial pathology in STZ-induced diabetic mice.

Mitochondrial fission and fusion occur in response to changing energy demands or to overcome unfavorable environments by allowing for the mixing of metabolites and mitochondrial DNA [21, 28]. Mitochondrial fission is necessary for mitochondrial renewal, redistribution, and proliferation; mitochondrial fission also serves as a preventive mechanism because it segregates damaged and dysfunctional mitochondria from the healthy mitochondrial network [30–33]. Mitochondrial fusion leads to the maintenance of the bioactivity of the mitochondrial network and is essential for cell survival and growth [34–36]. Autophagic degradation of damaged or redundant mitochondria is a necessary process to maintain a healthy mitochondrial pool in cells [27]. When damaged and fragmented mitochondria accumulate in cells without removal by autophagy, excessive generation of ROS and release of apoptogenic proteins such as Cyt C can induce cell injury [23]. Mitochondrial dynamics and quality control continue to occur in cells, and imbalances in these processes are associated with reduced mitochondrial function and the development of disease [28]. Indeed, mutations in OPA1, a profusion protein that causes hereditary optic neuropathies [37] and mutations in MFNs and other fusion molecules, affect the function of placental cells, skeletal muscle cells, vascular smooth muscle cells, and peripheral motor neurons [28]. Mutations in autophagy- (Atg-) related genes associated with neurodegenerative disease, infectious disease, and cancer have also been identified [38]. Furthermore, LC3 deficiency results in increased collagen deposition in the obstructed kidneys of UUO mouse models [39].

There is also a growing body of evidence indicating that abnormal mitochondria may be important in the development and progression of diabetic kidney disease [11, 27]. In 1991, Takebayashi and Kaneda reported, for the first time, the occurrence of dysmorphic mitochondria in the proximal renal tubular cells of type 2 diabetic patients with microalbuminuria [40]. More frequent mitochondrial DNA deletion and reduction in mitochondrial protein expression was also observed in the kidneys of diabetic mice [12]. Moreover, metabolomic analysis of the urine of patients with diabetic kidney disease supports that mitochondrial dysfunction is crucial for DKD [11]. Therefore, we focused on renal tubules in DKD. Because renal tubules rely on mitochondrial oxidative phosphorylation to meet the continuous requirements of ATP to facilitate their resorptive function, it is conceivable that any abnormalities in mitochondrial function or life cycle could act on the pathogenesis of diabetic tubulopathies.

We observed low expression of PGC1*α*, a master regulator of mitochondrial biogenesis and respiration, in human renal proximal tubular cells exposed to high-glucose concentrations (Figure 1) [41, 42]. Concurrently, renal proximal tubular cells exhibited increased expression of a mitochondrial fission protein (Drp1) but reduced expression of a mitochondrial fusion protein (Mfn1) and reduced autophagy (LC3) after exposure to a high-glucose environment (Figure 2). Similarly, mitochondrial homeostasis was disrupted, and PGC1*α* expression was decreased in the renal tubules of STZ-induced diabetic mice (Figures 6(f) and 7). We asked whether the enhancement of PGC1*α* activity could lead to the restoration of mitochondrial dynamics and quality control and compensate for the deleterious consequences of a diabetic environment, such as apoptosis or fibrosis. PGC1*α* activity is regulated through phosphorylation and deacetylation by AMPK and SIRT1, respectively [18]. We used AICAR and metformin, PGC1*α* activators that act through the phosphorylation of AMPK, to answer this question. As shown in Figure 4, increased PGC1*α* activity induced restoration of the mtDNA/gDNA ratio and ameliorated the alterations in the expression levels of Drp1, Mfn1, and LC3II proteins caused by high-glucose concentrations in renal proximal tubular cells. AICAR and metformin treatment effectively mitigated albuminuria and renal histopathology (Figure 6). Examination of the tubular cells of diabetic mice via electron microscopy showed that most of the mitochondria were short and fragmented; however, after AICAR or metformin treatment, their shapes became more normal (Figure 7). However, these results should be interpreted with caution, because we could not completely exclude the possibility that drugs to boost PGC1*α* have some off-target effects in dosage used in vivo. In addition, we could not assess the exclusive effect of these drugs on kidney tubule and mitochondria in animal model. Clearly, more studies are needed in this area.

Few studies have been performed on mitochondrial dynamics and quality control in kidney disease [20, 23, 39]. Brooks et al. reported fragmented mitochondria in an experimental study of ischemic-reperfusion injury and cisplatin-induced nephrotoxicity in mice and showed that the inhibition of the profission protein Drp1 attenuates tubular cell apoptosis [20]. Myo-inositol oxygenase, a tubular-specific enzyme, also appears to be linked with mitochondrial fragmentation and quality control in tubular injury in DKD [23].

The roles of renal tubules have been overlooked in chronic kidney diseases such as DKD for many years. In vitro and in vivo evidence indicate that a high-glucose environment causes renal tubular cells to secrete molecules related to proinflammatory, profibrotic, and angiogenic responses through toll-like receptors without advanced glomerulopathy [43–45]. Disruption of mitochondrial dynamics and homeostasis is a plausible mechanism by which diabetic tubulopathy is provoked by diabetic stimuli [23, 27]. In this regard, enhancement of mitochondrial biogenesis through PGC1*α* activation could provide a dependable approach for patients with DKD.

In conclusion, we demonstrated that enhancement of PGC1*α* activity prevents alterations in mitochondrial dynamics and quality control caused by high-glucose concentrations in renal tubular cells and causes a consequent decrease in the tubulopathy of the kidneys of diabetic mice.

## Supplementary Material

We performed flow cytometry analysis using MitoSox showing reduced level of mitochondrial ROS production by treatment of PGC1a activators under high glucose condition in HKC8 cells (Supplementary Figure 1). Supplementary Figure 2 provides high magnified images of kidneys (PAS and Masson's trichorme staining) from normal control, diabetic control, diabetes + metformin and diabetic + AICAR groups. Supplementary Figure 3 showed the results of western blot analysis suggesting restoration of phosphorylated and total PGC1a expression by treatment of AICAR or metformin in diabetic kidneys.
