# Supplementary material for: PGC1α Activators Mitigate Diabetic Tubulopathy by Improving Mitochondrial Dynamics and Quality Control
Source: J Diabetes Res. 2017 Mar 20;2017:6483572. doi: 10.1155/2017/6483572 (PMC5376939; doi:10.1155/2017/6483572)
Supplement: Supplementary file 1 — We performed flow cytometry analysis using MitoSox showing reduced level of mitochondrial ROS production by treatment of PGC1a activators under high glucose condition in HKC8 cells (Supplementary Figure 1). Supplementary Figure 2 provides high magnified images of kidneys (PAS and Masson's trichorme staining) from normal control, diabetic control, diabetes + metformin and diabetic + AICAR groups. Supplementary Figure 3 showed the results of western blot analysis suggesting restoration of phosphorylated and total PGC1a expression by treatment of AICAR or metformin in diabetic kidneys. [file 6483572.f1.pdf]

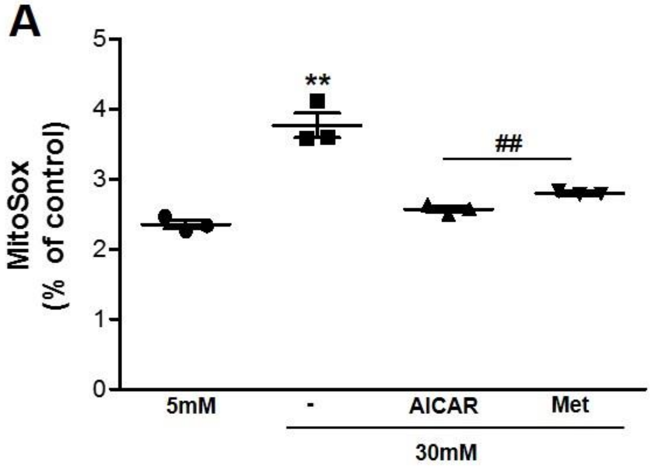


Supplementary figure 1. PGC1α activators decrease mitochondrial ROS production under high glucose condition. (A) flow cytometry of human renal proximal cells labeled with MitoSox following treatment with AICAR of Metfomin (*p < 0.01 vs 5mM, #p < 0.01 vs 30mM)

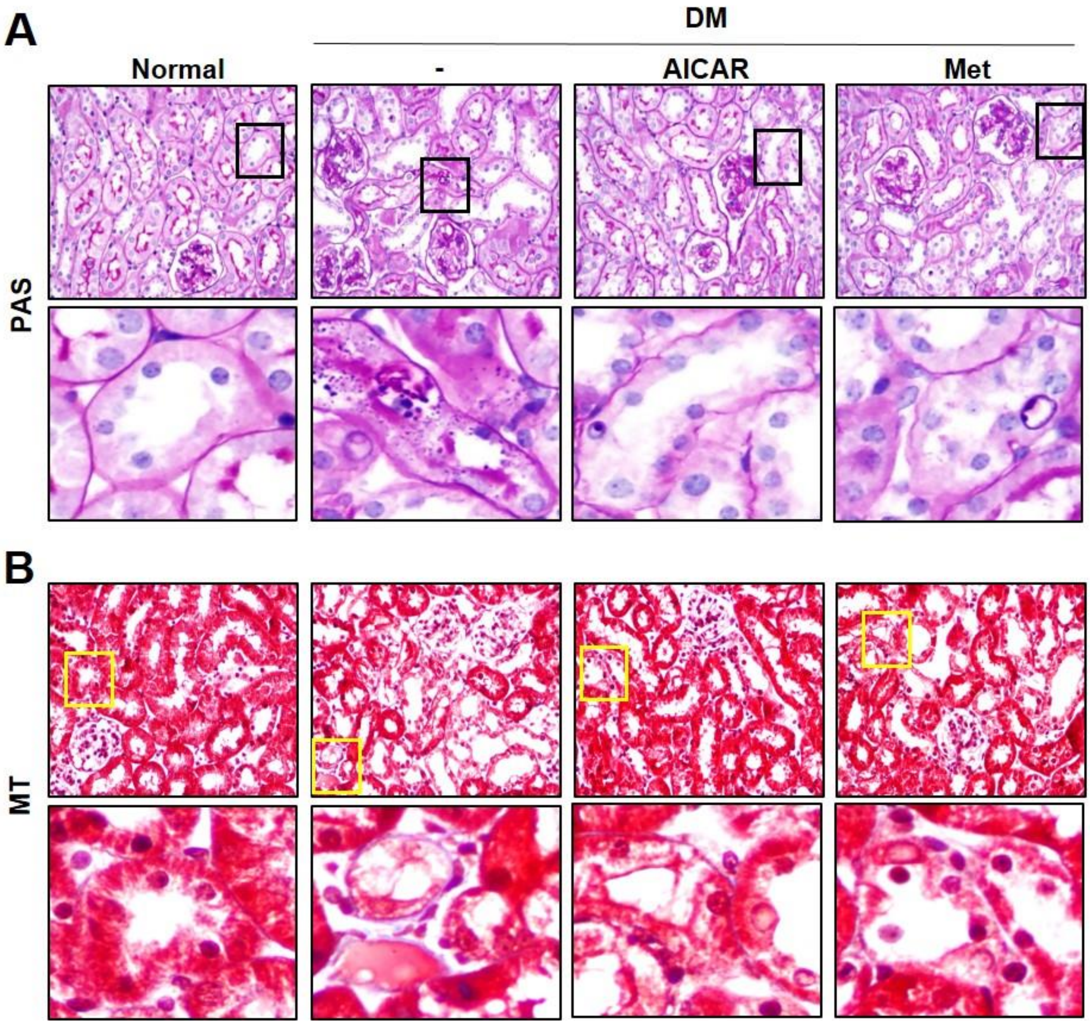


Supplementary figure 2. AICAR or metformin improve renal morphologic characteristics in kidneys of streptozotocin (STZ)-induced diabetic mice. (A) Tubular dilatation and tubular epithelial disruption were observed in the diabetic control group. AICAR and metformin restore tubular injury to some degree. (B) Representative of photographs of the Masson's trichrome-stained kidneys showed decrease of renal fibrotic lesions in AICAR and metformin treatment group compared with diabetic control group.

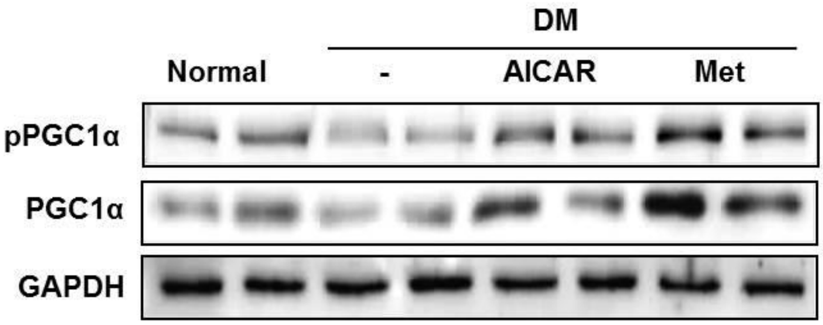


Supplementary figure 3. Administration of AICAR and metformin restore PGC1α activity in diabetic kidneys. Western blot analysis showed reduced expression of pPGC1α and PGC1α in diabetic kidneys, whereas their expression was recovered in the AICAR and metformin groups.
